# Predictive mathematical modeling of biomechanical behavior in all-on-4 implants design: effects of distal implant and occlusal load angulation using RSM based on FEA

**DOI:** 10.3389/fbioe.2025.1644776

**Published:** 2025-08-18

**Authors:** Fahri Murat, Sema Nur Sevinç Gül, Abdullah Tahir Şensoy

**Affiliations:** ^1^ Department of Mechanical Engineering, Faculty of Engineering and Architecture, Erzurum Technical University, Erzurum, Türkiye; ^2^ Department of Periodontology, Faculty of Dentistry, Atatürk University, Erzurum, Türkiye; ^3^ Faculty of Mechanical Engineering, Delft University of Technology, Delft, Netherlands; ^4^ Department of Oral and Maxillofacial Surgery, Erasmus University Medical Center, Rotterdam, Netherlands; ^5^ Department of Biomedical Engineering, Faculty of Engineering and Natural Sciences, Samsun University, Samsun, Türkiye

**Keywords:** All-on-4, implant angulation, occlusal load direction, finite element analysis (FEA), biomechanics, response surface method (RSM)

## Abstract

This study presents a predictive biomechanical modeling approach for optimizing distal implant placement in the All-on-4 treatment concept, with a focus on implant angulation and occlusal load direction. Finite Element Analysis (FEA) was integrated with Response Surface Methodology (RSM) to develop 15 simulation models based on a Central Composite Design, incorporating distal implant angulations of 15°, 30°, and 45°, and occlusal load directions in both sagittal and frontal planes (45°, 67.5°, and 90°). The maximum von Mises stress in cortical bone was selected as the response variable. Regression analysis revealed that the frontal load angle had the most significant effect on stress distribution, followed by implant angulation. The resulting second-order predictive model demonstrated a strong statistical fit (R^2^ = 93.39%, adjusted R^2^ = 81.49%). The lowest cortical stress (95.75 MPa) occurred at 15° implant angulation with 45° occlusal loading in both planes, whereas the highest stress (265.72 MPa) was recorded at 45° angulation with 90° frontal loading. Although reducing implant tilt generally decreases peri-implant stress, no universally optimal angle can be defined due to variability in biomechanical responses under different occlusal loading conditions. Clinically, optimizing cusp inclination and load direction in conjunction with implant positioning may enhance the biomechanical performance and long-term success of full-arch implant-supported prostheses.

## 1 Introduction

Dental implants have become a widely accepted treatment modality for the rehabilitation of missing teeth, aiming to restore both function and aesthetics. However, the success of implant therapy depends on a comprehensive understanding of the biological, mechanical, and psychological factors involved (Ozkir et al., 2013; Bhering et al., 2016). Tooth loss results in a wide range of complications, encompassing functional deficiencies, such as diminished masticatory performance, as well as psychological challenges secondary to compromised aesthetics. Therefore, a careful assessment of the indications and absolute contraindications for implant therapy is essential across all clinical scenarios, from single-tooth loss to full edentulism (Awasthi et al., 2019).

The All-on-Four treatment concept has revolutionized the rehabilitation of edentulous patients by enabling full-arch prosthetic restorations supported by only four implants. Two anterior implants are placed axially in the interforaminal region, while two posterior implants are strategically tilted to maximize the use of available bone and minimize the need for grafting procedures. The angulation of distal implants plays a critical role in biomechanical stress distribution, prosthetic longevity, and clinical success (Mocanu et al., 2020). Studies have shown that tilting distal implants between 30° and 45°, when accompanied by minimized cantilever lengths, can enhance stress distribution and improve clinical predictability (Mocanu et al., 2020; Kumari et al., 2020; Tribst et al., 2022; Santana et al., 2023). Specifically, 45° tilting may offer mechanical advantages, but only when cantilever lengths are kept short; otherwise, stress on both the bone and implant components tends to increase (Kumari et al., 2020; Tribst et al., 2022). Moreover, peri-implant stress has been shown to rise with increasing implant angulation, particularly in cases of poor bone quality or extended cantilevers (Kumari et al., 2020; Santana et al., 2023).

Chewing forces generate axial loads and bending moments, causing stress and deformation in both the implant and surrounding bone (Prados-Privado et al., 2020; Milone et al., 2022). Finite element studies also suggest that angled placement can lead to localized stress in peri-implant tissues (Anitua et al., 2022; Liu et al., 2022). Previous studies have consistently demonstrated that oblique loading induces higher stress concentrations compared to vertical loading, particularly in the cortical bone surrounding distal implants (Kilic and Caglar, 2024; Mahantshetty et al., 2021). Various implant configurations, such as tilted versus straight implants and standard versus short implants, have been investigated in this context. Notably, the use of tilted implants has been associated with increased stress in the peri-implant bone (Bhoi et al., 2023). Implant placement location also impacts stress distribution, with more posterior placement potentially preventing crestal bone resorption but increasing stress on prosthetic components (Gönül et al., 2022). Similarly, Ceddia et al. found that angled placement in low-density bone with thin cortical walls increases stress around the implant (Ceddia et al., 2024). However, the impact of implant angulation and its interaction with the occlusal loading on primary stability, especially as measured by the Implant Stability Quotient (ISQ), is still not well understood.

In recent years, there has been an increasing use of finite element analysis (FEA) and response surface methodology (RSM) in tandem. In this context, it can be considered as a significant instrument in the optimization of implant angulation in both the buccolingual and anteroposterior directions. FEA provides a three-dimensional analysis of the stress distribution at different implant angles and configurations. RSM is used to determine the ideal implant angle by statistically modelling the effect of multiple variables. The employment of these methodologies enables the biomechanical consequences of angulation of distal implants in All-on-4 prostheses to be evaluated with greater precision and predictability (Bukhari et al., 2024; Freitas et al., 2021). In this context, determining the ideal angulation for distal implants in anteroposterior direction in All-on-4 dental implant design is of great importance for prosthesis success and patient satisfaction. The integration of finite element analysis and response surface methodology facilitates the development of more personalized and scientifically substantiated treatment strategies within this domain (Bukhari et al., 2024; Freitas et al., 2021).

In contrast to traditional two-dimensional (2D) modelling, the integration of three-dimensional (3D) modelling based on medical imaging modalities such as computed tomography and magnetic resonance imaging has significantly advanced the precision of FEA in dental implant research. These imaging techniques enable accurate reconstruction of patient-specific anatomical structures, forming the basis for biomechanical simulations. In the context of optimizing implant angulation, particularly in complex systems such as All-on-4 prostheses, this patient-specific modelling enables a more realistic evaluation of stress and strain distributions under physiological loading conditions. Furthermore, combining FEA with RSM improves the ability to statistically evaluate and optimize multiple variables simultaneously. Consequently, the integration of imaging-based modelling, numerical simulation, and statistical optimization supports not only implant design and surgical planning, but also the development of evidence-based and customized treatment strategies (Prządka et al., 2025; Brachet et al., 2023).

To the best of our knowledge, no study in the literature has investigated the combined effects of implant angulation and occlusal loading on dental biomechanics. Therefore, the primary objective of this study is to determine the optimal occlusal load direction and distal implant angulation within the All-on-4 treatment concept using a hybrid methodology that integrates FEA and RSM. The study aims to develop a mathematical model that captures the relationship between these input parameters and cortical stress response. The ultimate goal is to minimize stress concentrations in peri-implant bone under functional loading, thereby enhancing biomechanical performance and supporting improved clinical outcomes.

## 2 Materials and methods

**FIGURE 1:**
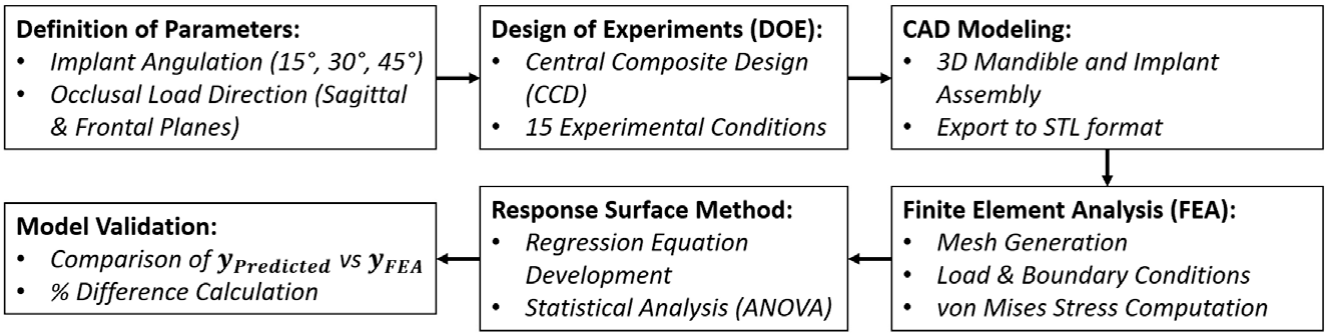
Workflow diagram summarizing the integration of RSM and FEA in the experimental design and biomechanical analysis process.

This study employed a computational workflow integrating FEA and RSM to evaluate the biomechanical behavior of distal implants in a full-arch All-on-4 rehabilitation scenario. The influence of implant angulation and occlusal load direction on peri-implant stress distribution was systematically assessed using simulation-based modeling. The workflow diagram followed in this study is given in Figure 1.

### 2.1 Three-dimensional model generation

**FIGURE 2:**
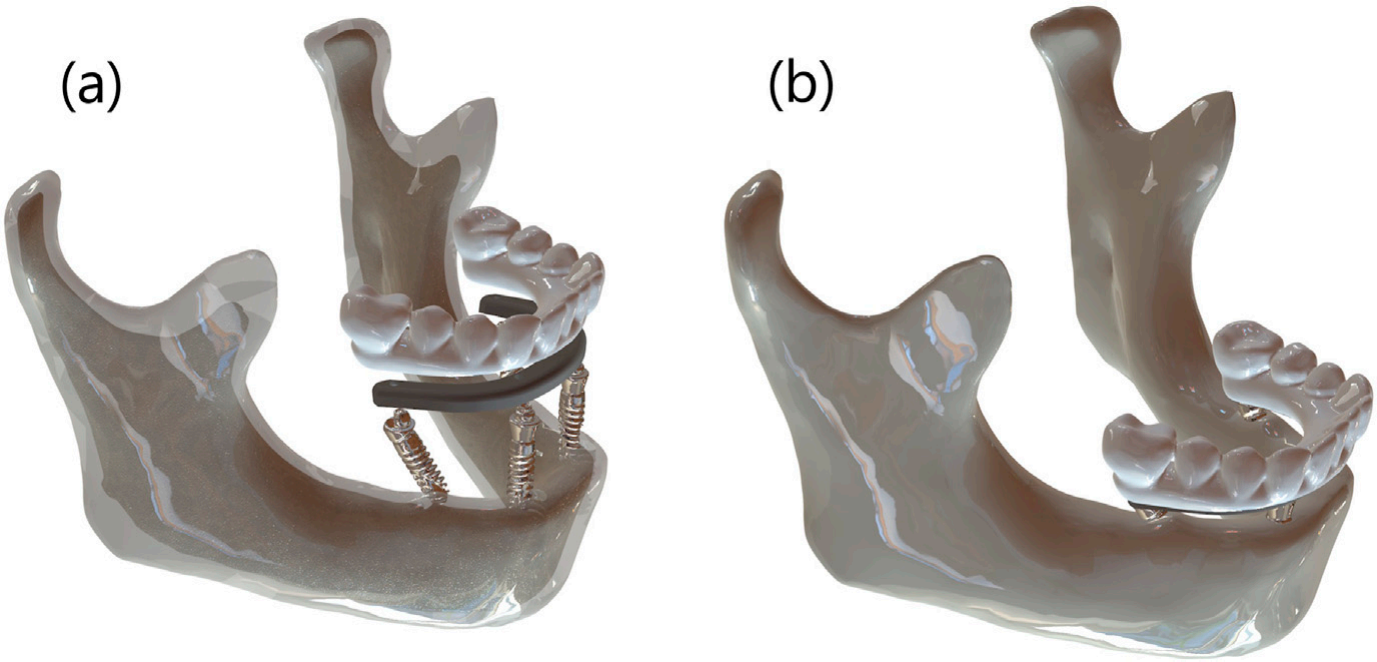
**(a)** Segmented 3D model showing cortical-trabecular differentiation with implant, bar, and prosthesis components, and **(b)** fully assembled atrophic mandible model.

A 3D finite element model representing a fully edentulous human mandible rehabilitated using the All-on-4 protocol was developed in SolidWorks 2024 (SolidWorks Corporation, Concord, MA, United States). Due to its high mechanical load exposure, the posterior implant site was designated as the region of interest in the simulations. The mandibular geometry was reconstructed based on previously acquired computed tomography (CT) data. A uniform cortical bone thickness of 2 mm was modeled, enclosing a cancellous core representative of trabecular bone structure. Figure 2 illustrates the model development process, including healthy and atrophic mandibular forms, segmentation of cortical and trabecular bone, and the integration of implant, bar, and prosthetic components.

**FIGURE 3:**
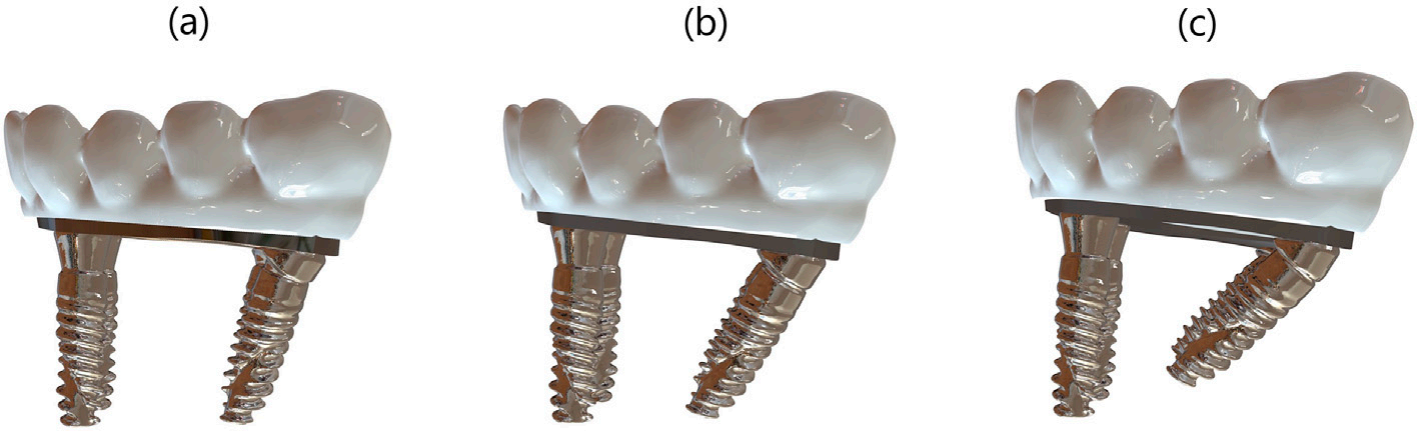
**(a)** 15°, **(b)** 30°, and **(c)** 45° angled distal implant configurations showing the corresponding bar and prosthetic superstructure in the All-on-4 model.

Titanium dental implants (Nobel Active, 4.3 mm diameter × 13 mm length; Nobel Biocare AB, Zurich, Switzerland) were virtually inserted into the mandibular model. Two anterior implants were placed vertically in the canine region, while the distal implants were inserted with angulations of 15°, 30°, and 45° relative to the occlusal plane. A distal cantilever of 5 mm was maintained in all configurations. All components were exported as STL files for simulation and meshing processes. Figure 3 shows prosthetic bar and crown configurations for the three implant tilt conditions (15°, 30°, and 45°).

All materials were considered homogeneous, isotropic, and linearly elastic. The mechanical properties assigned to the model components were derived from validated literature and are presented in Table 1.

**TABLE 1:** Material properties in the finite element model.

Component material (ref)	Elastic modulus (GPa)	Poisson’s ratio
Cortical bone (Sensoy et al. 2021)	13.70	0.30
Trabecular bone (Türker et al., 2019)	1.37	0.30
Titanium (implants) (Türker et al., 2019)	115	0.35
Ti-6Al-4V alloy (bar) (Liu et al., 2019)	110	0.33
Feldspathic porcelain(prosthesis) (Kilic and Doganay, 2020)	82.8	0.35

### 2.2 Boundary conditions

**FIGURE 4:**
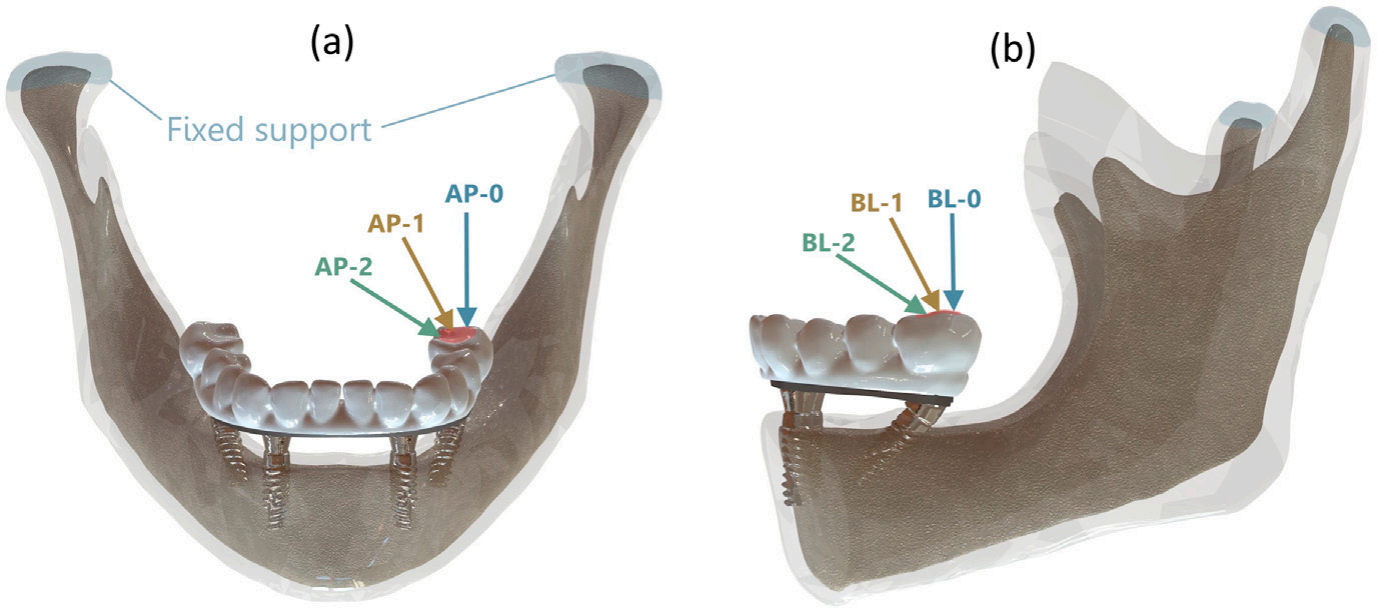
**(a)** Load angles in Sagittal plane with reference to anteroposterior (AP) direction, **(b)** Load angle in Frontal plane with reference to buccolingual (BL) direction.

To replicate functional loading scenarios, a static occlusal force of 200 N was applied to the distal prosthetic molar crown in all models, representing average chewing force. Load direction was systematically altered across both the sagittal and frontal planes at angles of 45°(AP-2, BL-2), 67.5°(AP-1, BL-1), and 90°(AP-0, BL-0) to assess biomechanical response under varied occlusal vectors. Implant tilt was independently varied at 15°, 30°, and 45° to observe interactive effects on peri-implant bone stress. Figure 4 depicts the complete 3D model assembly and the applied boundary and loading configurations for mechanical analysis.

To standardize all boundary conditions, the condylar surfaces were constrained in all degrees of freedom and ideal osseointegration was assumed between bone and implant interfaces. These settings were consistent across the entire simulation matrix, which was generated using Central Composite Design (CCD) principles.

### 2.3 Finite element analysis

All STL models were imported into ANSYS 2024R2 (Ansys Inc., Canonsburg, PA, United States) for meshing and finite element analysis. First-order tetrahedral elements were selected to discretize complex anatomical and prosthetic geometries. A preliminary convergence study was conducted to determine the ideal mesh density, balancing result accuracy and computational efficiency.

Mesh quality metrics for each configuration are summarized in Table 2. The number of nodes ranged from 1.10 to 1.23 million depending on implant angulation, and total element counts ranged from approximately 719,000 to 827,000. Element skewness remained below 0.29 for all configurations, and average element quality was maintained at 0.78.

**TABLE 2:** Table showing mesh skewness and number of elements according to distal implant angle.

Distal implant angle	15	30	45
Number of nodes	1235916	1103561	1126055
Number of elements	826239	718687	747814
Skewness	0.29	0.29	0.29
Element quality	0.78	0.78	0.78

**FIGURE 5:**
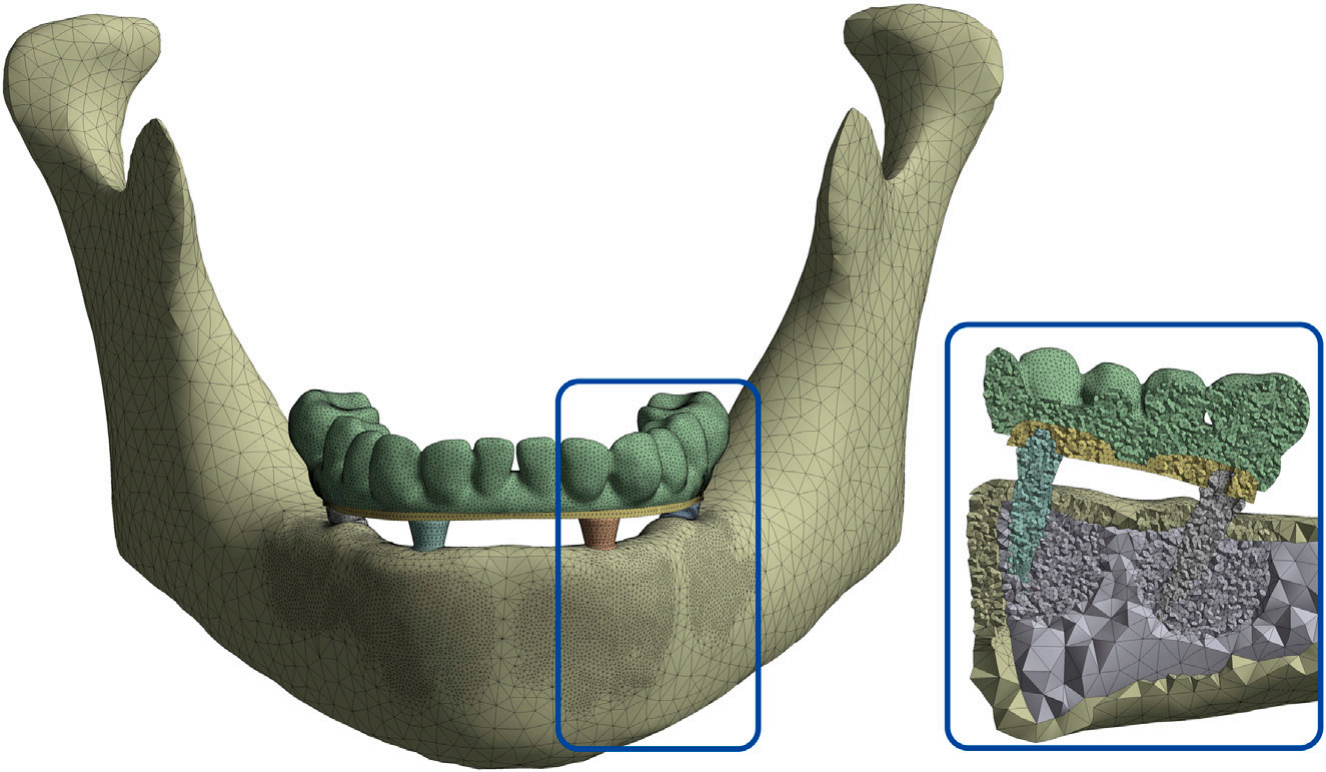
Cross-sectional view of the meshed mandibular model demonstrating element distribution across cortical and cancellous bone, implants, bar, and prosthetic components.

Component-specific mesh sizes were defined as follows: 3 mm for cortical and trabecular bone, 0.5 mm for implants and bar structures, yielding a global average element size of 1.3 mm. A cross-sectional view of the meshed model is illustrated in Figure 5, demonstrating the application of the mesh process and confirming the consistency of element distribution across the structure, which is critical for ensuring the validity of the finite element simulations.

### 2.4 Response surface modeling

This study employed a quantitative approach to investigate the effects of various input parameters on cortical stress levels. A multiple regression analysis was conducted using MINITAB statistical software (Minitab 22.0, Minitab Inc., State College, PA, United States) to assess the relationship between the dependent variable (y: cortical stress) and independent variables ( $x_{1}$ : Distal Implant Angle, $x_{2}$ : Load Angle in Sagittal Plane with reference to AP direction, and $x_{3}$ : Load Angle in Frontal Plane with reference to BL direction), including their interactions. Response Surface Method (RSM) based on central composite design (CCD) was used as the experimental design method. The parameters and levels are given in Table 3. The runs were randomly assigned, and 1 base block and 15 base runs were created. In the experimental design, a total of 15 points were utilized, consisting of 8 cube points, 1 center point within the cube, 6 axial points, and no center points in the axial configuration.

**TABLE 3:** Design Parameters and their levels for CCD.

Parameters	Definitions	Levels		
		−1	0	1
$x_{1}$	Distal Implant Angle	15	30	45
$x_{2}$	Load Angle in Sagittal Plane with reference to AP direction	45	67.5	90
$x_{3}$	Load Angle in Frontal Plane with reference to BL direction	45	67.5	90

A second-degree regression model has been formulated to establish a relationship between the response values obtained and the input parameters:

$$y=\beta_{0}+\sum_{i=1}^{k}\beta_{i}X_{i}+\sum_{i=1}^{k}\beta_{\text{ii}}X_{i}^{2}+\sum_{i=1}^{k-1}\sum_{j=i+1}^{k}\beta_{\text{ij}}X_{i}X_{j}+\varepsilon$$ (1)

In the equation, $\beta_{0\;}$ denotes the constant coefficient, $\beta_{i}$ signifies the coefficients for the linear terms of the regression model, $\beta_{\text{ii}}$ indicates the coefficients for the quadratic terms, $\beta_{\text{ij}}$ represents the coefficients for the interaction terms, $X_{i}$ refers to the specified independent variables (factors of the experimental design), and ε symbolizes the error term. Based on Equation 1, the regression model developed for the response parameter, which is the maximum equivalent von Mises stress in cortical bone, using the MINITAB statistical software (Minitab 22.0, Minitab Inc., State College, PA, United States) is shown in Equation 2:

$$y=-267+3.27x_{1}+4.03x_{2}+6.04x_{3}-0.\;0767x_{1}^{2}-0.0073x_{2}^{2}-0.0088x_{3}^{2}+0.0270x_{1}x_{2}+0.0250x_{1}x_{3}-0.0591x_{2}x_{3}$$ (2)

Equation 2 represents the second-order polynomial regression model developed using Response Surface Methodology (RSM) to predict the maximum von Mises stress $\left(y\right.$ ) in the cortical bone as a function of three independent variables. Here, $x_{1}$ denotes the distal implant angulation (in degrees), $x_{2}$ corresponds to the occlusal load angle in the sagittal plane (anteroposterior direction), and $x_{3}$ refers to the occlusal load angle in the frontal plane (buccolingual direction). These three parameters were systematically varied in the experimental design to assess their individual and interactive effects on peri-implant stress responses. By incorporating linear, quadratic, and interaction terms, the regression model allows for a comprehensive prediction of cortical bone stress under different biomechanical configurations in the All-on-4 implant scenario.

Table 4 shows the combinations of 3 independent input variables and the corresponding output for each design point. As 3 independent variables have been selected, 15 design points have been suggested using CCD.

**TABLE 4:** CCD-based experimental design and the corresponding response values.

Exp. No	Implant angle $x_{1}$	Load angle in sagittal plane $x_{2}$	Load angle in frontal plane $x_{3}$	Maximum von mises stress $y_{FEA}$ [MPa]	Maximum von mises stress $y_{Predicted}$ [MPa]	% Diff^a^
1	15	45	45	95.748	100.76	4.98
2	15	45	90	235.23	216.30	8.75
3	45	45	90	265.72	280.29	5.20
4	30	67.5	90	237.84	236.29	0.65
5	30	45	67.5	189.87	203.80	6.84
6	45	90	90	237.35	232.61	2.04
7	15	90	45	150.61	136.31	10.49
8	15	90	90	119.24	132.17	9.78
9	15	67.5	67.5	136.99	154.53	11.35
10	45	45	45	143.68	131.00	9.68
11	30	90	67.5	208.96	197.73	5.68
12	30	67.5	67.5	208	204.46	1.73
13	45	90	45	183.87	203.00	9.42
14	45	67.5	67.5	234.71	219.87	6.75
15	30	67.5	45	159.44	163.72	2.62

^a^ $\%Diff=100\times \frac{\left|y_{predicted}-y_{FEA}\right|}{y_{predicted}}$

Table 4 summarizes the CCD-based experimental design alongside both predicted and simulated stress values. In this context, $y_{Predicted}$ represents the cortical von Mises stress values estimated by the second-order polynomial regression model developed through Response Surface Methodology (RSM), while $y_{FEA}$ indicates the corresponding values computed directly via Finite Element Analysis (FEA). The final column, labeled %Diff (Difference), quantifies the relative deviation between the predicted and actual results, calculated as the percentage difference between $y_{Predicted}$ and $y_{FEA}$ . This metric provides a direct measure of the model’s predictive accuracy. Lower % difference values reflect high consistency between the RSM model and FEA simulations, confirming the model’s robustness in capturing biomechanical behavior under varying implant and load configurations. Conversely, higher deviations may be attributed to localized stress peaks or nonlinearities not fully accounted for by the regression model. Overall, the % Difference values in Table 4 validate the adequacy of the RSM model for approximating cortical stress responses with reasonable precision.

The collected data were analyzed using RSM to develop a second-order polynomial regression model relating the input parameters to the output stress values. Statistical analysis, including Analysis of Variance (ANOVA), was conducted to evaluate the significance of individual factors and their interactions (Table 5).

**TABLE 5:** Analysis of Variance (ANOVA) results of the model and the pareto chart of the standardized effects.

Source	DF	Adj SS	Adj MS	F-value	P-value
Model	9	33844.2	3760.5	7.85	0.018
Linear	3	23924.4	7974.8	16.64	0.005
X1	1	10726.4	10726.4	22.39	0.005
X2	1	91.3	91.3	0.19	0.681
X3	1	13106.7	13106.7	27.36	0.003
Square	3	1521.8	507.3	1.06	0.444
X1*X1	1	765.1	765.1	1.60	0.262
X2*X2	1	34.9	34.9	0.07	0.798
X3*X3	1	51.1	51.1	0.11	0.757
2-Way Interaction	3	8397.9	2799.3	5.84	0.043
X1*X2	1	665.2	665.2	1.39	0.292
X1*X3	1	568.0	568.0	1.19	0.326
X2*X3	1	7164.8	7164.8	14.95	0.012
Error	5	2395.6	479.1		
Total	14	36239.7			

**FIGURE 6:**
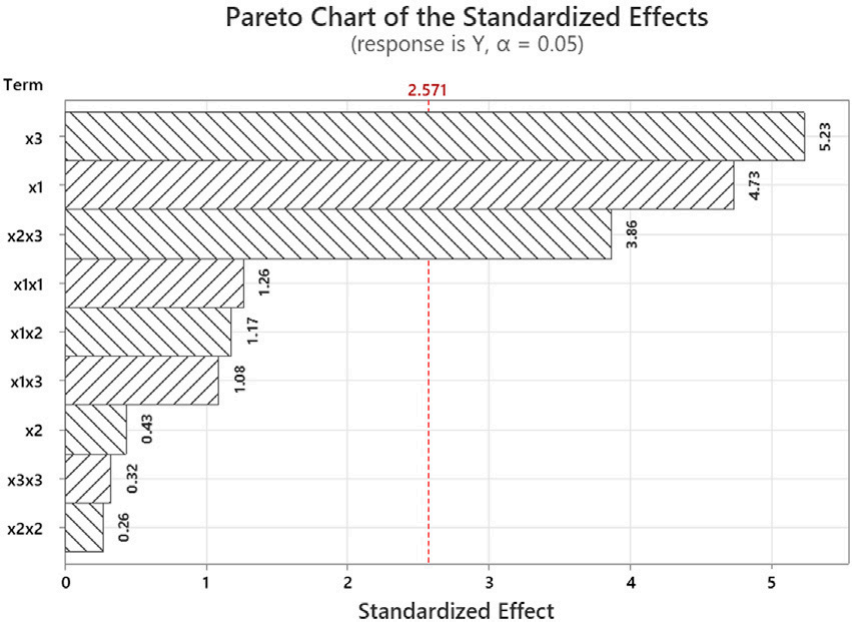
Pareto chart of the standardized effects.

Based on the model outputs, a Pareto chart of standardized effects was generated to determine the relative importance of each main factor and their interactions on the von Mises stress. The standardized effects provided a quantitative measure of each term’s contribution to the variation in the response (Figure 6).

## 3 Results

**FIGURE 7:**
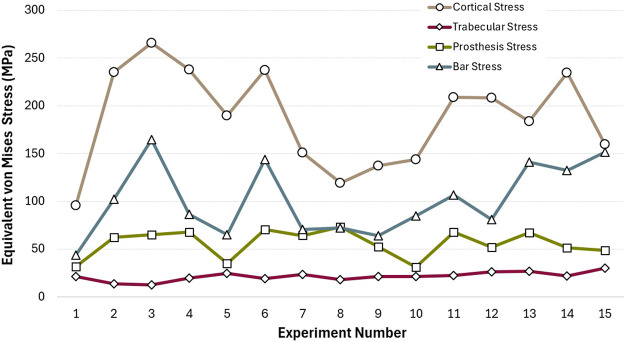
Equivalent von Mises stress values observed in cortical bone, trabecular bone, prosthesis, and bar components across all experimental configurations.

In this study, the biomechanical effects of different distal implant angulations and occlusal load directions were assessed using finite element analysis. Equivalent von Mises stress distributions for cortical bone, trabecular bone, the prosthetic framework, and the bar across all 15 experimental configurations are presented in Figure 7. Among these structures, cortical bone consistently exhibited the highest stress values, reaching a maximum of 265.72 MPa (Experiment 3) and a minimum of 95.75 MPa (Experiment 1). This indicates that implant angulation and load direction have a profound influence on cortical stress response.

The prosthetic superstructure experienced moderate stress levels, with peaks around 70.75 MPa (Experiment 6) and lows near 31.13 MPa (Experiment 10), indicating that certain angulation combinations may transmit more stress to the prosthetic components. Notably, bar stress demonstrated considerable variability, with a minimum of 43.53 MPa (Experiment 1) and a maximum of 164.38 MPa (Experiment 3). This fluctuation mirrors the pattern seen in cortical stress, highlighting a potential biomechanical coupling between these two components.

**FIGURE 8:**
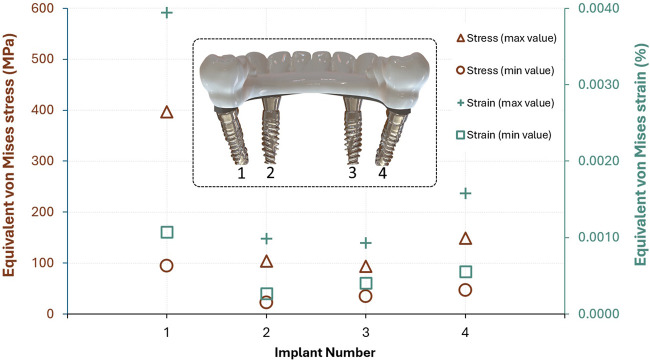
Maximum and minimum von Mises stress (MPa) and strain (%) values observed in each implant of the All-on-4 configuration (Implant numbers are shown from the posterior view of the lingual side).

Figure 8 illustrates the maximum and minimum equivalent von Mises stress and strain values for each implant in the All-on-4 configuration. The results reveal that Implant 1, located in the posterior region and likely subjected to sagittal oblique loading and higher cantilever forces, exhibited the highest biomechanical demands. It recorded a peak stress of 397.04 MPa and a maximum strain of 3,940 µɛ, substantially higher than the other implants. In contrast, Implant 3 demonstrated the lowest stress and strain values, with a maximum stress of 93.32 MPa and strain of 930 µɛ, suggesting a more favorable load distribution.

The relatively high minimum stress values in Implants 1 and 4 also indicate that these locations experience consistent mechanical loading, possibly due to their role in supporting distal occlusal forces. On the other hand, Implant 2 had the lowest minimum stress (22.42 MPa) and strain (266µɛ), indicating a less critical biomechanical role in the system. These findings suggest that posterior implants, particularly Implant 1, are subjected to greater stress concentrations, underlining the importance of angulation and prosthetic design in load mitigation strategies.

**FIGURE 9:**
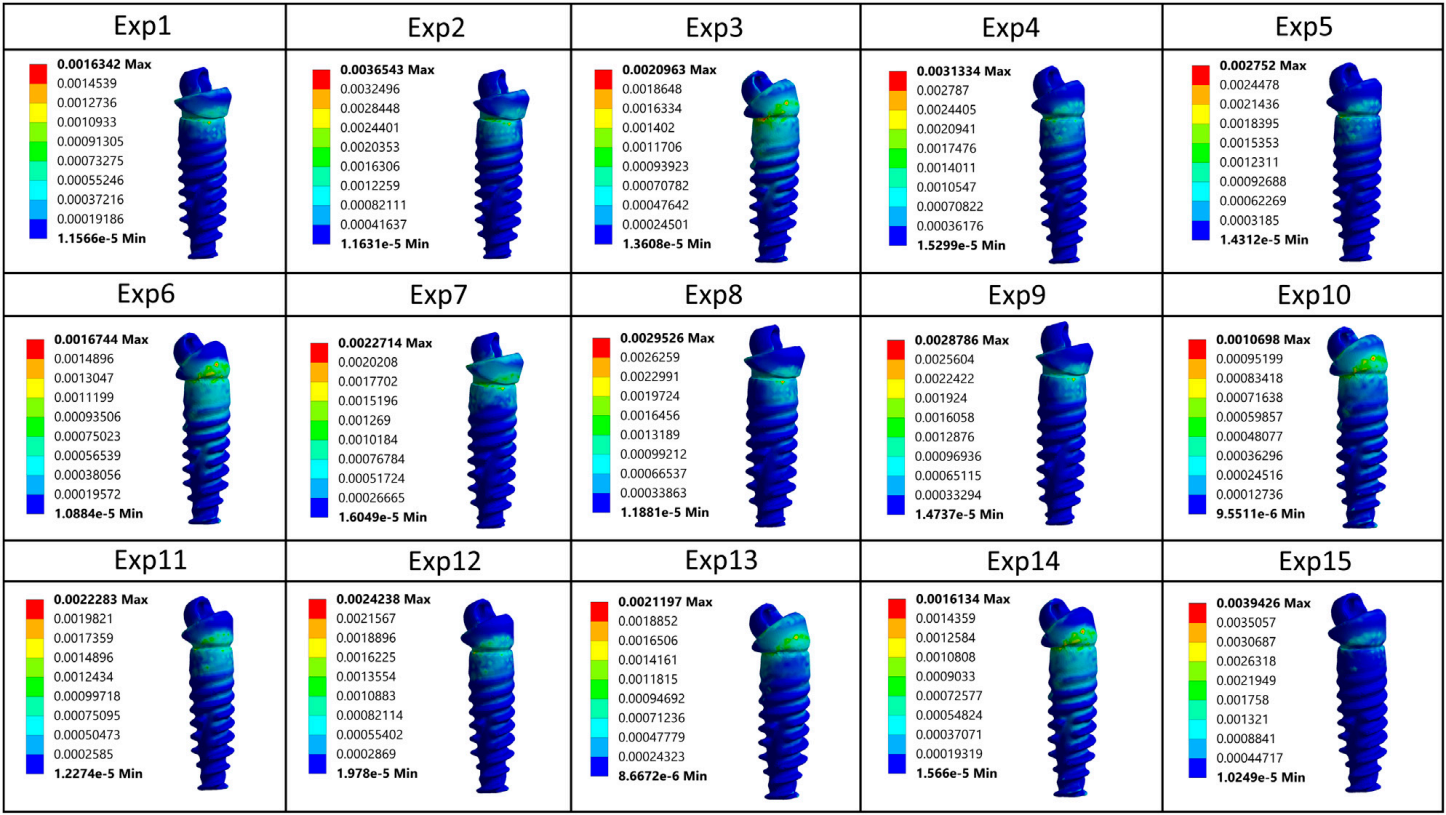
Comparative von Mises strain patterns on the distal implant across all 15 simulated loading configurations.

A comparative inspection of von Mises strain distributions across the distal implant, as visualized in Figure 9, reveals marked variability in biomechanical response depending on the implant-load configuration. Notably, in Experiments 2 and 15, elevated strain concentrations near the implant neck were recorded as 3,654 µɛ and 3,940 µɛ, respectively, the latter representing the maximum among all cases. This suggests an increased likelihood of microstrain accumulation in these configurations, potentially predisposing surrounding cortical bone to overload and adverse remodeling.

In contrast, more favorable strain profiles were observed in Experiments 1, 10, and 14, which maintained lower peak strain values within the range of 1069–1634 µɛ, indicative of more efficient stress transfer and mechanical equilibrium. Across all simulations, strain intensity consistently attenuated toward the apical region, reaffirming the expected gradient behavior of axial and oblique loads in osseointegrated implants. The colorimetric variation across models underscores the sensitivity of peri-implant strain to subtle changes in both implant inclination and loading vector orientation, further emphasizing the necessity for individualized biomechanical optimization in All-on-4 rehabilitations.

**FIGURE 10:**
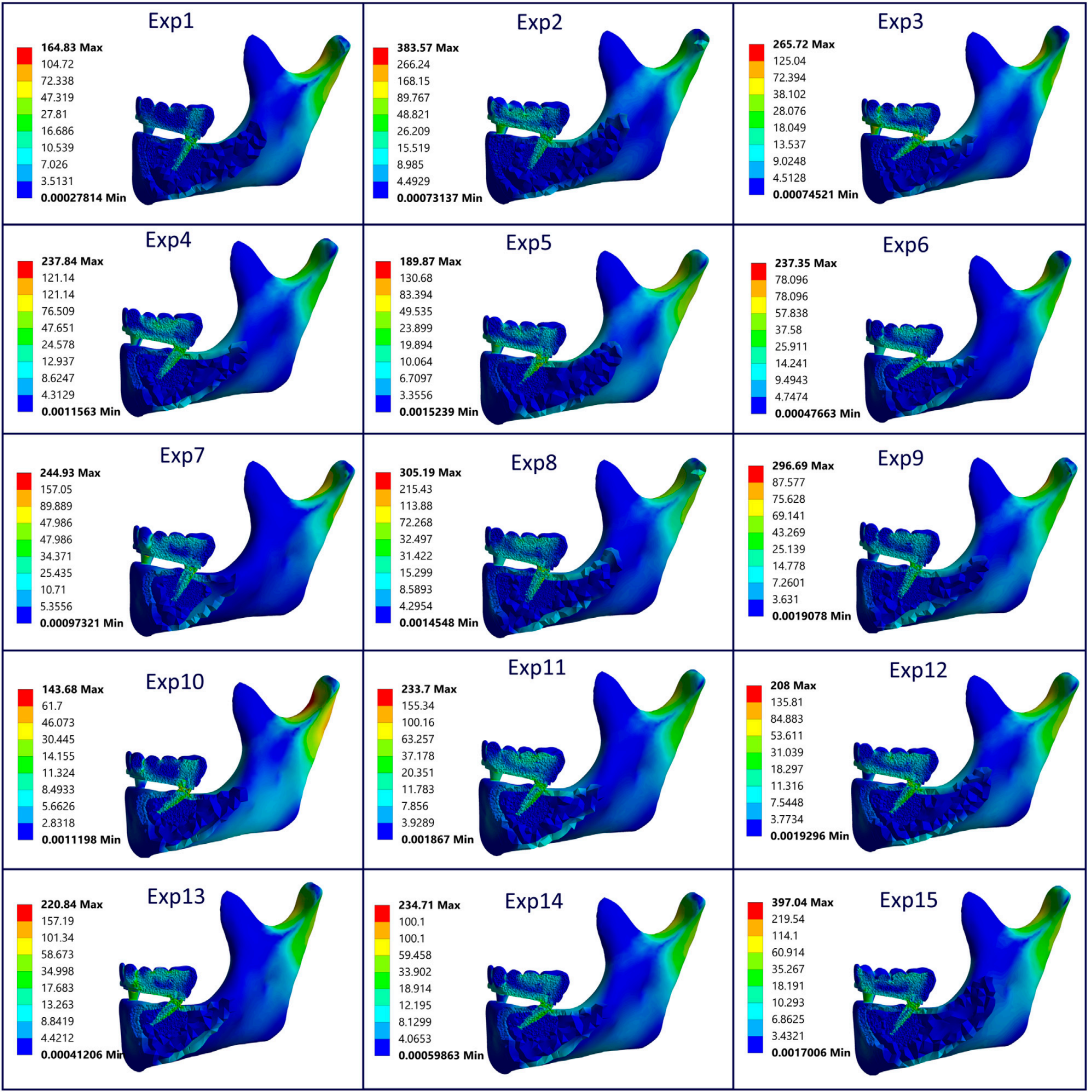
Sectional von Mises stress distribution across cortical/trabecular bone, implants, bar, and prosthesis for all models, highlighting component-specific stress concentrations under varying angulation and load conditions (All units are in MPa).

Figure 10 illustrates von Mises stress distributions across all major components—cortical and trabecular bone, implants, bar, and prosthetic superstructure—based on sectional views from each experimental model. The highest stress concentrations are consistently observed around the implant neck, particularly in Experiments 2 and 15, where values exceed 380 MPa. These results suggest suboptimal biomechanical performance under specific angulation and load scenarios. In contrast, Experiments 1 and 10 show more balanced stress dispersion with lower peak values, reflecting improved load transfer. Overall, stress is most pronounced in the upper implant and prosthetic connection regions, diminishing apically and into the trabecular bone.

**FIGURE 11:**
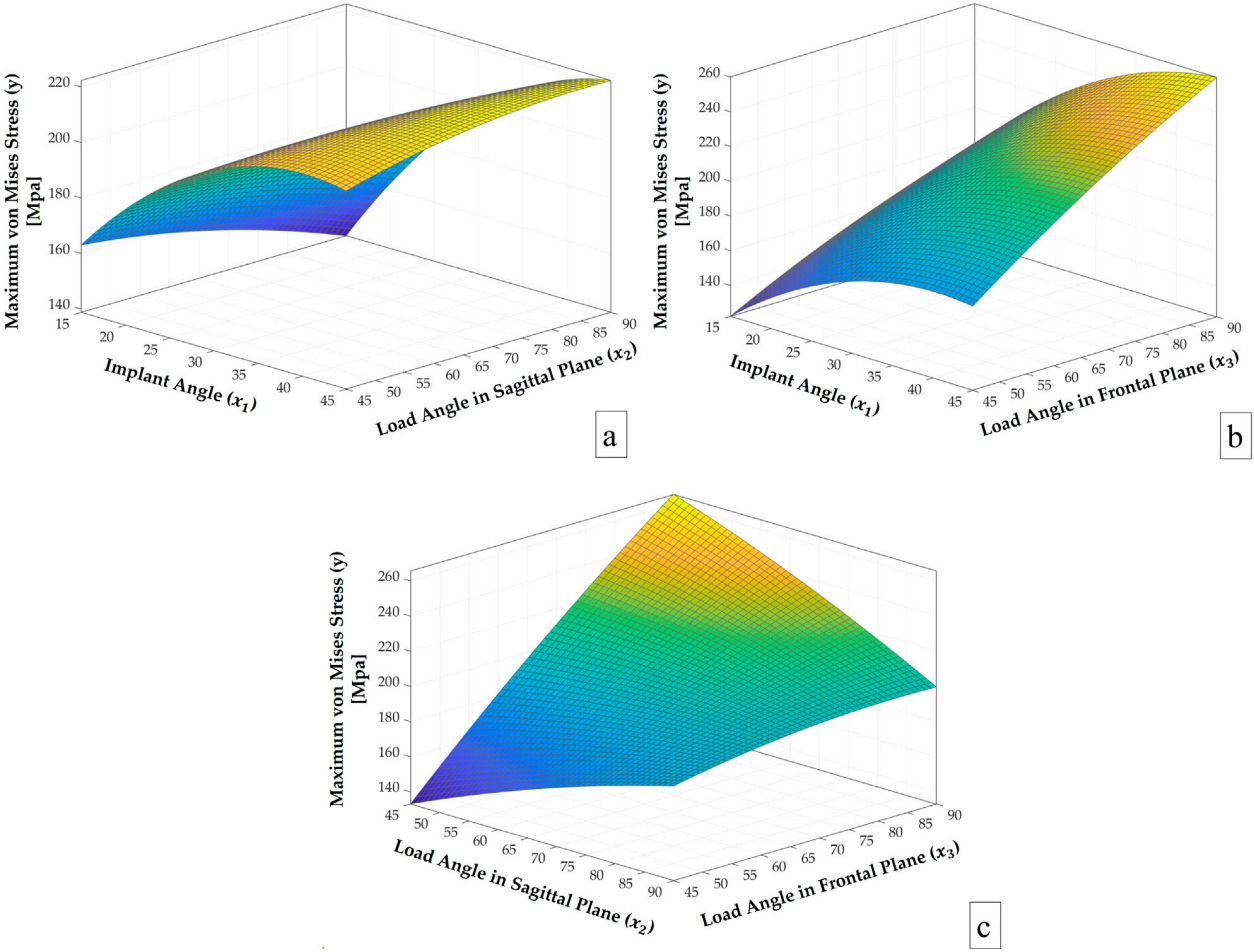
Response surface plots of y vs. **(a)** $x_{1}$ and $x_{2}$ , **(b)** $x_{1}$ and $x_{3}$ , **(c)** $x_{2}$ and $x_{3}$ .

In the response surface plots (Figures 11a–c), one input parameter in Equation 2 was kept constant at its mean value to enable visualization in three-dimensional space, while the other two were varied. In Figure 11b, for instance, the highest cortical stress occurred at x_1_ = 45°, x_2_ = 67.5 (mean), and x_3_ = 90. The plots indicate that cortical stress tends to rise as implant angulation increases and loading becomes more oblique, with the effect being particularly evident in the frontal plane. The combination of steep frontal loading and high implant angles produced the highest stress levels, whereas more vertical implant configurations were associated with lower stresses. Additionally, when x_2_ was fixed at its upper value, changes in x_3_ produced relatively minor variations in cortical stress. In contrast, when x_2_ was fixed at its lower value, increases in x_3_ led to a dramatic increase in stress levels. This suggests that the influence of the frontal load angle becomes particularly critical under sagittal oblique loading conditions.

The results of the analysis of variance (ANOVA) shown in Table 5 revealed that the overall regression model was statistically significant (p = 0.018, F = 7.85), indicating that the combination of variables used in the model had a meaningful effect on the response variable. Among the linear terms, x_1_ (implant angle) and x_3_ (load angle in frontal plane) exhibited statistically significant effects on the maximum von Mises stress, with p-values of 0.005 and 0.003, respectively. In contrast, x_2_ (load angle in the sagittal plane) showed no significant influence (p = 0.681). The squared terms (x_1_^2^, x_2_^2^, x_3_^2^) collectively did not show a statistically significant contribution (p = 0.444), suggesting limited curvature in the response surface within the tested range. Among the two-way interaction terms, the interaction between x_2_ and x_3_ was statistically significant (p = 0.012), while the other interaction effects (x_1_x_2_ and x_1_x_3_) were not significant (p = 0.292 and p = 0.326, respectively). Despite some statistically insignificant higher-order terms in the model, the adjusted mean square error (479.1) is considered acceptable, as the model yields a maximum prediction error of only 11.35% across 15 observations. The model demonstrated a strong fit to the data, with an R^2^ value of 93.39%, indicating that it explains a large proportion of the variance in the response, while the adjusted R^2^ value of 81.49% confirms its robustness after accounting for the number of predictors. Overall, the findings indicate that the implant angle and the load angle in the frontal plane are the primary contributors to stress distribution, and that the interaction of sagittal load angle with the frontal load angle also plays a notable role.

## 4 Discussion

The All-on-4 dental implant concept is designed to maximize bone-to-implant contact while minimizing cantilever length. Achieving proper distal implant angulation is essential for maintaining implant stability and peri-implant bone health. This study aimed to determine the best distal implant orientation under both vertical and oblique loading conditions through a hybrid computational approach that integrated FEA and RSM.

The stress distribution patterns observed in this study highlight the biomechanical sensitivity of cortical bone to variations in implant angulation and loading direction. The findings revealed that both implant angulation (x_1_) and occlusal load direction particularly the frontal plane angle (x_3_) significantly influence stress distribution patterns. The statistical analysis confirmed that the load angle in the frontal plane (x_3_) was the most influential variable, exhibiting the highest standardized effect at 29.83%. This was followed by the implant angle (x_1_) with a contribution of 26.96%, while the sagittal load angle (x_2_) showed no significant linear effect. Among the interaction terms, x_2_x_3_ had a notable influence at 14.65%, whereas x_1_x_2_ and x_1_x_3_ contributed smaller effects of 6.19% and 1.83%, respectively. These findings align with the Pareto principle, suggesting that a limited number of key parameters are responsible for the majority of the variation in the stress response. This suggests that deviations in frontal load direction may exert greater biomechanical influence on peri-implant stress than sagittal shifts alone. However, the significant interaction between x_2_ and x_3_ implies that sagittal loading should not be overlooked, especially when combined with steep implant angulation. These results are consistent with previous FEA-based studies reporting increased stress concentrations under oblique loading and tilted implant configurations (Ulu et al., 2024; Li et al., 2015; Kaptı et al., 2024), reinforcing the mechanical disadvantage of steep distal tilting when load vectors deviate from the vertical axis. Conversely, more favorable stress distributions were observed when both implant angle and load direction were aligned closer to the vertical, supporting the biomechanical benefit of axial force transfer. Pareto chart further demonstrated that a small number of variables account for most of the variation in cortical stress, emphasizing the importance of implant and load orientation optimization in full-arch restorations.

The markedly higher von Mises stress values in cortical bone, peaking at 265.72 MPa, underscore its critical role in load transfer within the implant-supported system. These findings align with previous research showing that stress in cortical bone increases significantly with greater abutment angulation and non-axial loading, often concentrating around the implant neck and crestal bone region (Uppalapati et al., 2023; Almeida et al., 2015; Behnaz et al., 2015). In contrast, the relatively low and stable stress levels in trabecular bone suggest a buffering role, with less susceptibility to biomechanical alterations, as similarly reported by studies using finite element analyses (Vijapure et al., 2019; Leblebicioğlu Kurtuluş et al., 2022). This differential response between bone types reinforces the importance of optimizing implant placement to minimize cortical stress and preserve long-term osseointegration.

The variability in von Mises strain distributions observed across simulations emphasizes the critical influence of implant-load configurations on peri-implant biomechanics in All-on-4 rehabilitations. Elevated strain concentrations localized at the implant neck reached up to 3,654 µɛ and 3,942 µɛ in Experiments 2 and 15, respectively, approaching or exceeding thresholds associated with pathological bone remodeling and microdamage (Tribst et al., 2022; Durkan et al., 2020). In contrast, Experiments 1, 10, and 14, which exhibited lower strain magnitudes, demonstrated more favorable load transmission and mechanical equilibrium, consistent with evidence suggesting that optimized implant inclinations and minimized cantilever lengths reduce strain and the risk of biomechanical overload (Kumari et al., 2020; Takahashi et al., 2010). These findings reinforce the necessity of strategic implant placement and loading control to preserve cortical bone integrity and prevent adverse adaptive responses over time.

The findings of this study highlight that a distal implant angulation of 15° mostly yielded the lowest von Mises stress values, identifying it as biomechanically optimal within the tested parameters. This aligns with previous studies reporting increased peri-implant stress with greater angulations; for instance, Sayed and Mohamed noted a progressive stress rise from 15° to 45°, while other research showed significantly higher stress accumulation at 30° and 45°, especially in cortical bone and prosthetic connector areas (Sayed and mohamed, 2024; Begg et al., 2009; Pellizzer et al., 2011). The results presented in Table 4 and Figure 7 support these findings, as Experiment 3 exhibited peak stress level exceeding 250 MPa at crestal cortical bone areas commonly affected by angulated implants, particularly under sagittal oblique loading. Conversely, according to the stress distribution patterns observed in Figure 10, Experiments 1 and 10 displayed more uniform stress dispersion, suggesting superior biomechanical performance in these configurations.

The results indicate that angulation in the frontal plane tends to have a more detrimental effect on stress distribution. However, as observed in Table 4 (Exp. 1 and 10), a 45° angulation in the sagittal plane demonstrates a beneficial influence on stress values, which can be attributed to the reduction in the moment generated by occlusal loading. These results highlight the influence of implant angulation and prosthetic design on stress distribution patterns and their potential role in maintaining peri-implant bone stability over time. The findings support the notion that individualized treatment planning may contribute to more favorable biomechanical conditions, as suggested in previous studies (Kim et al., 2025).

However, some clinical studies have reported no statistically significant differences in outcomes between tilted and axially placed implants. For instance, a randomized controlled trial found no difference in implant success between 0° and 30° angles after 24 months (Badr et al., 2023). Similarly, a clinical investigation in severely atrophic mandibles reported high survival rates and minimal bone loss (0.85 mm at 12 months), regardless of implant tilt (Agliardi et al., 2010). These favorable clinical findings contrast with the current FEA results, which showed increased stress concentrations in tilted configurations. This discrepancy may stem from biological adaptation mechanisms *in vivo*, such as bone remodeling, which are not fully captured in computational models. Variations in anatomical conditions, loading protocols, or healing capacities also contribute to this divergence.

The stress behavior observed in the prosthetic superstructure and bar components highlights the biomechanical significance of implant angulation and prosthetic configuration in influencing load distribution. Moderate yet variable stress values within the framework suggest that specific angulation and loading orientations can meaningfully alter the force transmission across the prosthesis. These findings are supported by finite element studies reporting that mesially or distally tilted implants, particularly under oblique loading or in the presence of misfits, induce increased stress in both the bar and surrounding structures (Tribst et al., 2022; Caldas et al., 2018). The similar stress patterns seen in both the bar and the cortical bone suggest that these two components are biomechanically linked. This connection becomes even more important in full-arch prostheses, where tilting the implants without adjusting the cantilever length can lead to increased stress (Kumari et al., 2020; Tribst et al., 2022; Takahashi et al., 2010). Although angled implants are often used in clinical practice to reduce cantilever length and mechanical overload, this study kept the cantilever length constant at 5 mm in all models to better understand the specific effects of implant and load angulation. This controlled approach allowed a more precise assessment of how angular changes alone impact stress behavior. These insights reinforce the importance of individualized implant positioning, load control, and framework design to mitigate biomechanical overload and ensure long-term prosthetic success.

Shahriari et al. (2019) also investigated stress patterns in All-on-4 designs using FEA and found that tilted implants may reduce bone loss risk compared to parallel configurations, referencing the mechanostat theory. Discrepancies between their findings and the present study may result from differences in model assumptions, most notably boundary conditions involving different combinations of occlusal loading angles across different planes, as well as from variations in cortical thickness, and other geometric parameters that can significantly influence FEA outcomes.

The results of the current study clearly indicate that greater implant inclination and sagittal oblique loading produce the highest cortical stress, especially near the crestal bone (Exp. 3 Table 4; Figure 10). These findings align with studies showing that as abutment or implant angulation increases, particularly under non-axial loads, stress concentrations intensify around the implant neck and adjacent cortical bone (Uppalapati et al., 2023; Leblebicioğlu Kurtuluş et al., 2022). Notably, the dominant impact of the frontal load angle (x_3_) on stress outcomes, followed by implant angle (x_1_) and sagittal load angle (x_2_), suggests that lateral forces are biomechanically more disruptive. This is consistent with findings indicating that oblique loading may produce higher stress in cortical bone than axial forces, especially with high abutment angulations (Almeida et al., 2015; Vijapure et al., 2019). These results reinforce the necessity for clinicians to minimize the sagittal load angle especially at the implant angle of 15° to maintain optimal implant orientation to reduce stress-related risks and ensure long-term prosthetic success (Inci et al., 2024).

As with all FEA-based investigations, this study includes several simplifying assumptions that impact the realism of the simulations. Material properties were modeled as homogeneous, isotropic, and linearly elastic, and geometric and loading conditions were standardized. While these choices improve computational efficiency, they may fail to capture the anatomical and mechanical complexity of real clinical scenarios (Trivedi, 2014). These limitations reduce the generalizability of findings and necessitate cautious interpretation. Moreover, discrepancies across similar studies indicate that optimal implant angulation is shaped by multiple interrelated factors, including patient anatomy, bone quality, implant design as well as age-related factors and systemic conditions. Potential sources of error in the present simulation include the use of generalized material properties from the literature, idealized boundary conditions such as full osseointegration and fixed condyles, and limitations related to mesh generation. While the mesh quality met established standards, localized inaccuracies may still influence peak stress values. Although such simplifications can affect the absolute magnitude of calculated stresses, their consistent application across all models helps preserve the internal validity and reliability of comparative outcomes.

While finite element analysis offers valuable insights into stress distribution and implant biomechanics, this study is also limited by the absence of experimental (*in vitro* or *ex vivo*) and clinical validation. Numerical models cannot fully reflect biological variability or long-term functional outcomes. Previous studies have reported inconsistencies between FEA predictions and clinical results, often due to factors such as healing processes, bone remodeling, or patient-specific anatomical variability (Trivedi, 2014; Vaidyanathan and Fathima, 2022). Consequently, future research should aim to validate FEA findings through mechanical testing and longitudinal clinical data to enhance translational accuracy and practical applicability.

Despite these limitations, the combined use of RSM and FEA provided a powerful tool for multi-factorial biomechanical optimization. The response surface model enabled efficient prediction of stress outcomes without the need for excessive simulation runs. However, as with all computational models, experimental and long-term clinical validation is essential before translating these findings into routine clinical practice. Future studies should consider patient-specific modeling and dynamic loading conditions to further refine biomechanical risk assessments.

## 5 Conclusion

By simulating 15 implant-load configurations, the biomechanical behavior of peri-implant bone was quantified, and the most critical factors influencing stress distribution were identified and some concluding remarks were obtained as follows:

• Frontal load direction (x_3_) was the most influential variable on cortical stress, followed by implant angulation (x_1_).
• The lowest stress occurred at 15° implant angulation under vertical loading; the highest was observed at 45° angulation with sagittal oblique loading (stress >260 MPa).
• A significant interaction between sagittal and frontal load angles (x_2_·x_3_) indicated compounded stress under combined oblique vectors.
• Peak stress consistently localized at the implant neck and crestal cortical bone, regardless of configuration.
• RSM enabled accurate prediction of stress responses, reducing dependency on repeated FEA runs.

The results of the study highlight the pivotal importance of minimizing implant tilt and avoiding oblique loading, especially in scenarios with minimal implant tilt, to effectively reduce peri-implant stress and enhance biomechanical stability in All-on-4 protocols. Although reducing implant tilt generally decreases peri-implant stress, no definitive implant tilt angle can be universally recommended due to the variability in biomechanical responses under different occlusal loading conditions. Future research should incorporate variations in bone quality and cortical thickness to enhance the clinical applicability and predictive accuracy of biomechanical models. While the proposed method presents a promising strategy for treatment planning, further validation through clinical data and incorporation of patient-specific variables remain essential.

## Data Availability

The original contributions presented in the study are included in the article, further inquiries can be directed to the corresponding author.
